# The Effect of the Solution Flow and Electrical Field on the Homogeneity of Large-Scale Electrodeposited ZnO Nanorods

**DOI:** 10.3390/ma17061241

**Published:** 2024-03-08

**Authors:** Yanmin Zhao, Kexue Li, Ying Hu, Xiaobing Hou, Fengyuan Lin, Jilong Tang, Xin Tang, Xida Xing, Xiao Zhao, Haibin Zhu, Xiaohua Wang, Zhipeng Wei

**Affiliations:** 1 State Key Laboratory of High Power Semiconductor Lasers, Changchun University of Science and Technology, 7089 Wei-Xing Road, Changchun 130022, Chinahouxiaobingdavid@163.com (X.H.); linfengyuan_0116@163.com (F.L.);; 2 Zhongshan Institute of Changchun University of Science and Technology, Zhongshan 528437, China; 3 School of Optoelectronics, Beijing Institute of Technology, Beijing 100081, China; 4 Shandong North Optical Electronic Co., Ltd., Taian 271000, China; 5 Northeast Industry Group Co., Ltd., Changchun 130212, China

**Keywords:** zinc oxide nanorod arrays, large-scale, electrodeposition, homogeneous nanorod arrays

## Abstract

In this paper, we demonstrate the significant impact of the solution flow and electrical field on the homogeneity of large-scale ZnO nanorod electrodeposition from an aqueous solution containing zinc nitrate and ammonium nitrate, primarily based on the X-ray fluorescence results. The homogeneity can be enhanced by adjusting the counter electrode size and solution flow rate. We have successfully produced relatively uniform nanorod arrays on an 8 × 10 cm^2^ i-ZnO-coated fluorine-doped tin oxide (FTO) substrate using a compact counter electrode and a vertical stirring setup. The as-grown nanorods exhibit similar surface morphologies and dominant, intense, almost uniform near-band-edge emissions in different regions of the sample. Additionally, the surface reflectance is significantly reduced after depositing the ZnO nanorods, achieving a moth-eye effect through subwavelength structuring. This effect of the nanorod array structure indicates that it can improve the utilization efficiency of light reception or emission in various optoelectronic devices and products. The large-scale preparation of ZnO nanorods is more practical to apply and has an extremely broad application value. Based on the research results, it is feasible to prepare large-scale ZnO nanorods suitable for antireflective coatings and commercial applications by optimizing the electrodeposition conditions.

## 1. Introduction

Antireflective coatings (ARCs) play a crucial role in enhancing the optical absorption or light extraction efficiency of optoelectronic devices, such as solar cells, solar energy heat collectors, LDs, LEDs, PDs, and others. Numerous efforts have been made to explore high energy conversion efficiency, as a significant portion of incident or emitted light is lost due to surface reflection. In crystalline silicon solar cells, for instance, approximately 36% of the incident or emitted light is directly reflected, leading to substantial energy utilization loss [1,2]. To address this issue, it is important to enhance the light trapping or output by minimizing undesirable surface reflection losses through the use of ARCs. Achieving an effective antireflective effect involves satisfying two conditions: firstly, antireflective materials should be transparent, avoiding additional optical absorption, and secondly, the refractive index (n) of the materials should be between that of air and the device materials. Conventional ARCs reduce reflectivity by utilizing destructive interference with the waves reflected from the interface of the ARC with the top of devices and their surroundings. Typically, these ARCs are thin films with a quarter-wavelength thickness and a certain refractive index (RI) to minimize the reflectivity. But for ARC thin films, the antireflection effect works only at a single wavelength and at the normal incidence of light. In contrast to bulk materials, nano-scale structures (e.g., NWs, NRs, nanotips, nanocolumns, nanopores) can increase the incident light path and enhance the light absorption. These structures can be equivalent to a gradient change in the refractive index in the direction of incident light according to the formula of effective medium approximation, and a good light trapping effect can be obtained by adjusting the morphology of the nanostructure [3]. This property attracts the exploration of nanostructures as effective ARCs. In this work, we pay attention to the large-scale ZnO NRs fabricated. 

Common materials for ARCs include MgF_2_, CaF_2_, SiN, ZnS, and ZnO. Among these materials, zinc oxide is a promising candidate for antireflective coatings due to its abundance in the Earth’s crust and suitability for various manufacturing processes. ZnO is cost-effective, environmentally friendly, and non-toxic. Moreover, it possesses good transparency, a wide band gap (Eg~3.37 eV at 300 K), an appropriate refractive index (n~1.9), and the ability to form textured coatings via anisotropic growth. Furthermore, ZnO offers several advantages. It is a semiconductor with a large exciton binding energy (60 meV), and high-quality films and nanostructures can be prepared at relatively low temperatures (less than 700 °C). It is more resistant to radiation damage and has a high mechanical strength, with a Young’s modulus of 25–150 GPa and a high fracture strain property. Additionally, it exhibits a thermal expansion coefficient from 5 × 10^−6^ to 8 × 10^−6^/°C within the temperature range of 25–400 °C. ZnO is a favorable material for nanostructure functionality [4,5,6]. Among all kinds of nanostructures, quasi-one-dimensional ZnO nanostructures, such as nanorods (NRs) and nanowires (NWs), have garnered significant attention for their potential applications in high-technology fields, including electronics and optoelectronics. These structures find applications in light-emitting diodes [7,8], photodetectors [9,10], photodiodes [11], gas sensors [12], and solar cells [13]. For ARC applications, ZnO nanorod arrays, featuring a high length-to-diameter ratio, a high surface area-to-volume ratio, and a unique morphology, as well as perfect crystalline structures, are expected to significantly improve light absorption and are considered a new generation of ARCs. ZnO nanorod (NR) arrays offer several advantageous properties, including good transparency for the solar spectrum and a subwavelength structure size, as well as an appropriate refractive index of ~2, leading to continuously varying refractive index profiles in the arrays [14,15]. Thus, they can effectively suppress surface reflection and enhance light coupling through phenomena such as zero-order grating characteristics or the moth-eye effect [16,17]. Nanostructures of ZnO with an RI profile between air and bulk ZnO are one of the most promising candidates [18,19,20] for ARCs, which are physically and technically compatible with the window layer. Consequently, these characteristics suggest that ZnO NRs are emerging as promising materials for solar cell antireflective coatings and solar thermal selective surfaces [21,22,23,24,25,26], demonstrating remarkable prospects. 

In the solar cell region, their effectiveness as antireflective coatings (ARCs) is supported by the related research. Aé, L. demonstrated that the short-circuit current of a solar cell can realize a relative increase of nearly 6% before and after the deposition of ZnO NRs [18]. Furthermore, Bai, A. and Tang, Y. fabricated a ZnO NR ARC on Cu(In,Ga)Se_2_ thin-film solar cells, successfully reducing the weighted reflectance from 8.6% to 3.5% [27]. In order to realize practical application, deposition at a large scale is necessary and valuable, paving the way for the industrialized production of ZnO NR ARCs. For production, various methods have been employed to prepare ZnO NRs, for example, chemical vapor deposition [28,29], the vapor transport method [30], thermal evaporation [31], and the vapor–liquid–solid technique [32]. Compared to these methods, electrodeposition is simpler and more compatible with low-temperature substrates due to its moderate operating temperature [33,34,35,36,37,38]. It is also an energy-efficient and cost-effective method for applications in large-scale fabrication in industry thanks to the use of aqueous solutions in an open atmosphere. But for large-scale electrodeposition, achieving homogeneity in the ZnO NRs is crucial yet challenging. Therefore, it is important to analyze and determine the factors affecting the homogeneity of deposition, facilitating the production of a large-area uniform ZnO NR ARC and aiding future industrialization efforts. Among the influencing factors for homogenous large-scale deposition, the solution flow and the electric field should play significant roles in zinc electrodeposition. The morphology appears to be susceptible to the flow field and also dependent on the flow pattern [39,40]. R.D. Naybour’s results have clearly shown that the hydrodynamic condition of the electrolyte is an important factor in controlling the morphology of the electrodeposits [41]. Naybour pointed out that changes in the surface morphology of electrodeposited zinc occur because there is a large amount of microturbulence in the electrolyte during deposition [42]. Milan M. considered that toroidal vortices and local fluctuations existed in the electrolyte, bringing a higher bulk concentration of the reacting species into regions of the electrode surface [42], thereby providing for faster local and spatially periodic growth of the deposit. These local concentration fluctuations could contribute to the local current density change [43], resulting in local growth rate differences.

In our experiments, in order to confirm the influence of the solution flow and electric field, ZnO NRs were deposited onto a cathode electrode from aqueous electrolyte solution; the ZnO deposition should also be affected by the solution flow. Besides random thermal motion, concentration gradient diffusion, and solution flow transportation, ions also are driven by the electric field between the counter and working electrodes in electrodeposition, which also will affect the deposition outcomes. Altering the shape of the counter electrode and the distance between the working and counter electrodes can adjust the electrical field distribution, thereby impacting the transportation behavior of the reacting species. Regarding the electrical field factor, we established the significant influence of the electrical field on the homogeneity of the ZnO NRs by merely changing the counter electrode size at a fixed distance. In situations with a small counter electrode, the electrical field balanced the solution flow factor in our electrochemical setup, resulting in relatively homogenous ZnO NR deposition when combined with stirring. Based on our research and tests, we successfully fabricated uniform and large-scale ZnO NRs (8 × 10 cm^2^) by optimizing the solution flow and electric field distribution.

## 2. Materials and Methods

In the deposition process, we immersed all samples in an aqueous solution containing 7 mM zinc nitrate (Zn(NO_3_)_2.6_H_2_O) and 5 mM ammonium nitrate (NH_4_NO_3_) (purity > 99.99%, purchased from Alfa Aesar, Haverhill, MA, USA), subjecting them to a potential of −1.4 V in potentiostatic mode. This electrochemical procedure employed a three-electrode configuration, incorporating a Pt counter electrode and a pseudo-reference electrode (IviumStat. H, Ivium, Eindhove, The Netherlands), both immersed in a large water bath to ensure optimal temperature control. The deposition temperature was meticulously maintained at 75 °C. To systematically explore the impact of the solution flow on the homogeneity of the ZnO nanorods (NRs), we deposited samples 1–3 onto fluorine-doped SnO_2_ (FTO) substrates. These substrates were strategically paired with a spacious counter electrode (12 × 12 cm^2^ Pt foil) under various conditions, including stirring at the center, no stirring, and stirring at the bottom, strategically positioned between the counter and working electrodes within the electrochemical cell. Additionally, we prepared the ZnO NRs in sample 4 utilizing a smaller counter electrode (5 × 6 cm^2^ Pt foil, centrally clipped onto 10 × 10 cm^2^ polycarbonate plates) without employing stirring. This approach was designed to isolate the influence of the electrical field, allowing for a comparative analysis against the results obtained using larger counter electrodes. It is noteworthy that these samples were deposited using a single-contact working electrode (top side). Subsequently, we cultivated relatively homogeneous ZnO NRs in sample 5 on FTO substrates that were pre-coated with a ~30 nm undoped ZnO film (i-ZnO/FTO). This innovative process involved utilizing both sides (left and right) of the working electrode and the smaller counter electrode, with consistent stirring. It is essential to highlight that all the substrates used in this study were 8 × 10 cm^2^ in size. Prior to the deposition process, each substrate underwent ultrasonic cleaning in acetone and ethanol, followed a thorough rinsing in Milli-Q water for 5 minutes at each stage. Subsequent to this meticulous cleaning procedure, the substrates were dried using nitrogen gas to ensure a pristine surface for the subsequent deposition steps.

Assessment of the homogeneity across all the samples was meticulously carried out employing a state-of-the-art Philips MagiX Pro wavelength-dispersive X-ray fluorescence (XRF) spectrometer (PANalytical B.V., Alemlo, The Netherlands). This cutting-edge instrument facilitated a detailed analysis by converting the detected Zn content in the ZnO nanorod (NR) region into the film thickness. This innovative approach enabled us to derive the homogeneity data by meticulously comparing the thickness variations across different regions within each sample. To delve even further into the structural characteristics, the morphology of the relatively homogeneous sample was subjected to rigorous scrutiny utilizing a Zeiss LEO 1530 scanning electron microscope (SEM, Zeiss, Oberkochen, Germany) equipped with a Gemini 4000 column. This advanced microscopic examination not only provided valuable insights into the structural uniformity but also offered a closer look at the surface characteristics of the ZnO nanorods. Such detailed observations are instrumental in comprehending the intricacies of the nanostructures and optimizing their performance in optoelectronic applications. Moving beyond structural analysis, we conducted room-temperature photoluminescence (PL) measurements for the aforementioned sample. These were meticulously recorded utilizing a He-Cd laser, employing an excitation wavelength of 325 nm. The resulting PL spectra serve as a crucial source of information concerning the optical properties and emission characteristics of the ZnO nanorods under ambient conditions. This spectral analysis is indispensable for understanding the electronic transitions within the material, shedding light on its photonic behavior. In addition to the PL studies, we ventured into an assessment of the reflectance spectra within the 400–800 nm wavelength range. This was achieved using a UV–vis–NIR spectrophotometer (UV 3600 Plus, Japan Shimadzu, Kyoto, Japan), coupled with an integrating sphere. The acquired reflectance spectra furnish valuable insights into the interaction of light with the ZnO-nanorod-coated substrates. Analyzing the reflectance characteristics is pivotal for gaining a comprehensive understanding of the optical performance and antireflective properties exhibited by the deposited ZnO nanorods in this specific spectral range. This multifaceted approach contributes to a holistic exploration of the material’s optical behavior, enriching the scientific discourse and paving the way for advancements in semiconductor optoelectronics.

## 3. Results and Discussion

Table 1 provides a comprehensive overview of the X-ray fluorescence (XRF) results for samples 1–3. The segmentation into Top (T), Middle (M), and Bottom (B) regions, along with the Left (L), Middle (M), and Right (R) regions within each sample, facilitates a detailed analysis of the spatial variations in ZnO content. An intriguing observation emerges from samples 1 and 2, where a discernible reduction in ZnO presence is noted in the central regions. This intriguing phenomenon suggests a potential disparity in the density or dimensions of the ZnO nanorods (NRs) within these areas, particularly when ZnO NRs are deposited under conditions involving central stirring or the absence of stirring. Our hypothesis is rooted in the notion that the solution flow dynamics contribute to the observed variations. Specifically, we postulate that the influx of reaction ions onto the substrate is less pronounced in the central region compared to the surrounding areas. This discrepancy in the ion concentration is likely a consequence of the solution’s intricate flow patterns during the deposition process. According to the following equations that were suggested by Izaki et al. [44,45],

Zn(NO_3_)_2_ <=> Zn^2+^ + 2NO_3_^−^ (1)

NO_3_^−^ + 2e + H_2_O → 2OH^−^ + NO_2_^−^ (2)

Zn^2+^ + 2OH^−^ <=> Zn(OH)_2_ (3)

Zn(OH)_2_ → ZnO + H_2_O (4)

**Figure 1:**
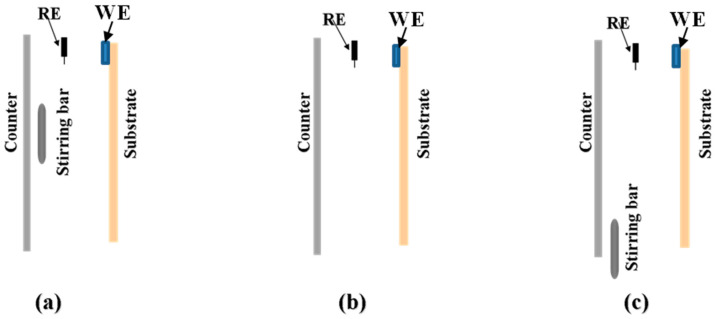
Schematic diagram of samples 1–3 deposited under stirring in the middle, without stirring, and stirring away from the center, which correspond to pictures (**a**–**c**), respectively. (RE: pseudo-reference electrode; WE: working electrode).

These reactions provide valuable insights into the complex processes occurring during ZnO NR deposition. Notably, we draw attention to the fact that the growth rate of NRs in the central region is anticipated to be slower. This deceleration can be attributed to the lower availability of reacting species reaching the substrate simultaneously. Since these reactions primarily unfold on the substrate’s surface during deposition, it is plausible that the solution in the central region readily exchanges with a more reacted solution than in the surrounding areas. To illustrate this concept, we present depositing schematic diagrams (Figure 1a,b), visually demonstrating the slower NR deposition rate in the central region under the conditions of central stirring or the absence of stirring. These schematics serve as a valuable tool for comprehending the dynamic interactions influencing ZnO NR growth across different regions within the samples. The spatial variations in the ZnO content observed in samples 1 and 2 align with our hypothesis of differential reaction ion availability being influenced by the solution flow dynamics. This nuanced understanding contributes to the optimization of ZnO NR deposition processes, offering insights that can enhance the uniformity and quality of nanorod structures. 

**Figure 2:**
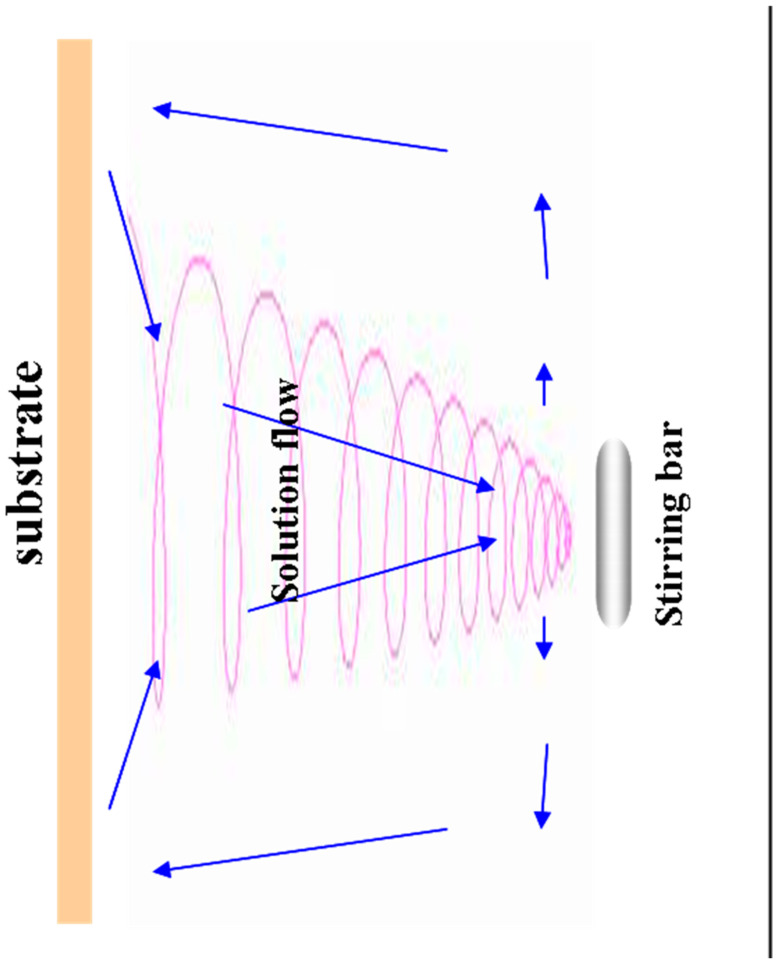
The schematic diagram of solution flow that was stirred with stirring bar located at center. (The direction of the arrow indicates the direction of liquid flow and exchange.)

In the deposition process of sample 3, a unique approach was implemented, with stirring taking place away from the center and the stirring bar strategically positioned at the bottom of the counter electrode, as illustrated in Figure 1c. Subsequent to the deposition, an in-depth X-ray fluorescence (XRF) analysis of sample 3 uncovered a compelling observation—a discernibly thinner ZnO layer in the Bottom (BM) region, as detailed in Table 1. This finding serves as a critical piece of evidence, further substantiating the hypothesis that the dynamic nature of the solution flow exerts a substantial influence on the homogeneity of the ZnO nanorod (NR) deposition. Upon a meticulous comparison of the XRF results between sample 1 and sample 3, a nuanced inference comes to light. It becomes evident that the solution in close proximity to the substrate, particularly facing the stirring center, exhibits a heightened susceptibility to exchange with the surrounding reacted solution, facilitated by the stirring action. This intricate interplay results in a fascinating helical structure-like contraction of the solution flow toward the stirring bar, a phenomenon visualized in Figure 2—a schematic diagram illustrating the dynamic solution flow under stirring conditions. The consequence of this dynamic behavior extends to local growth rate differences, particularly near the surface of the large-scale substrate. The fluctuation in the concentration of the reacting species within both the central and surrounding regions amplifies the heterogeneity in the growth rates of the ZnO nanorods. This localized growth rate difference is particularly pronounced in the vicinity of the stirring bar, contributing to the observed variations in the ZnO layer’s thickness in the BM region of sample 3. In essence, the stirring-induced solution flow dynamics play a pivotal role in dictating the homogeneity of the ZnO NR deposition, leading to nuanced localized growth rate differences. This newfound understanding holds significant implications for optimizing large-scale ZnO NR fabrication processes, enhancing the precision and uniformity of nanorod structures. 

Table 2 provides a detailed comparison of the X-ray fluorescence (XRF) results for samples 2 and 4, both deposited without stirring, utilizing large and small counters, respectively. Unlike sample 2, where a higher ZnO content was observed in the surrounding regions, sample 4 exhibited a more uniform ZnO distribution, showcasing a closer match between the central and surrounding areas. This notable uniformity is attributed to the variations in the electrical field distribution, influenced by the counter size. In previous studies, researchers like M.-M. Zhang and R.G. Reddy have simulated the electrical field distribution during electrodeposition in ionic liquids, demonstrating that the main electrical gradient exists around the edges of electrodes. Their modeling results indicate that the current density near the edges of the electrode is generally more intensive than elsewhere within the cross section [46]. Applying this concept to our experiments, the larger counter likely created a stronger electrical field around the edge of the working electrode, promoting enhanced ZnO deposition in the surrounding regions. Conversely, in the case of the smaller counter, the electrical field may have been weakened in the surrounding region near the substrate, effectively balancing the transportation of the reacting ions toward the substrate. Consequently, for sample 4, the growth rate of the nanorods (NRs) in the center approximates that of the surrounding regions. This intriguing observation underscores the importance of the counter size in controlling the electrical field and, subsequently, the homogeneity of ZnO NR deposition.

Based on the aforementioned details, sample 5 was meticulously prepared on i-ZnO/FTO with stirring conditions, utilizing Cu foils as a dual-sided working electrode to mitigate the observed ZnO gradient tendency in sample 4. To address the potential reduction from top to bottom, both sides of the Cu foils were employed during the nanorod (NR) electrodeposition. The normalized X-ray fluorescence (XRF) results in Table 3 reveal a less pronounced gradient from top to bottom in sample 5. This suggests that the observed gradient in sample 4, attributed to potential reduction, is mitigated or at least not significantly pronounced. The refined experimental setup emphasizes the role of the solution flow in influencing the homogeneity of ZnO NR deposition, offering valuable insights for optimizing large-scale fabrication processes. 

**Figure 3:**
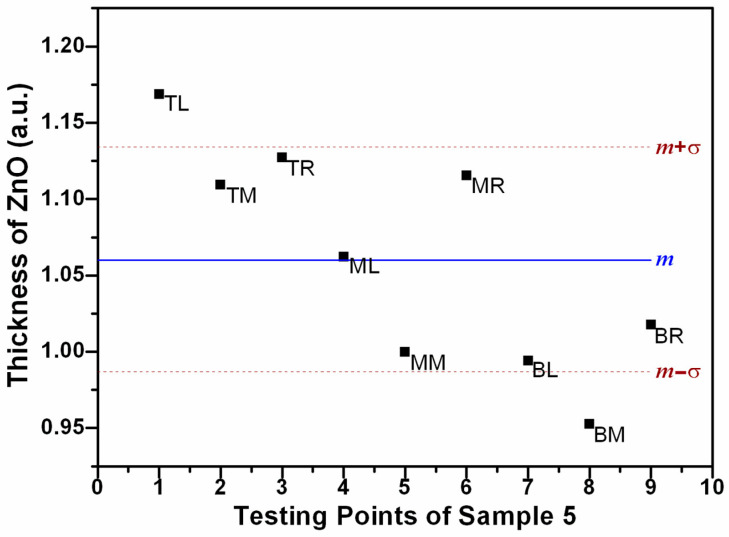
Normalized XRF results of sample 5 with an average thickness of ZnO m = 1.06 (a.u.) and a standard deviation σ = 0.07 (a.u.).

**Figure 4:**
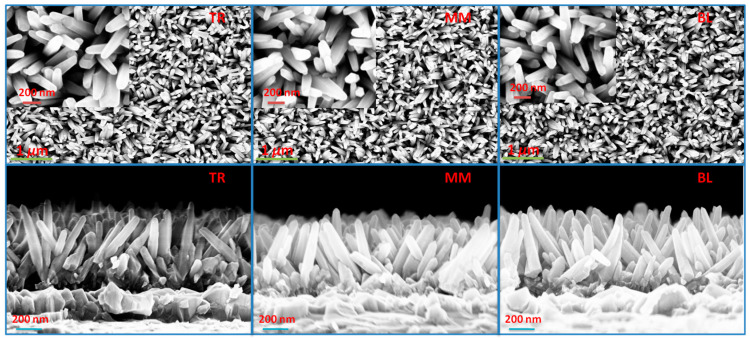
Top-view and cross-section SEM micrographs of ZnO nanorods in the TR, MM, and BL sections of sample 5. Enlarged images are shown in the insets.

**Figure 5:**
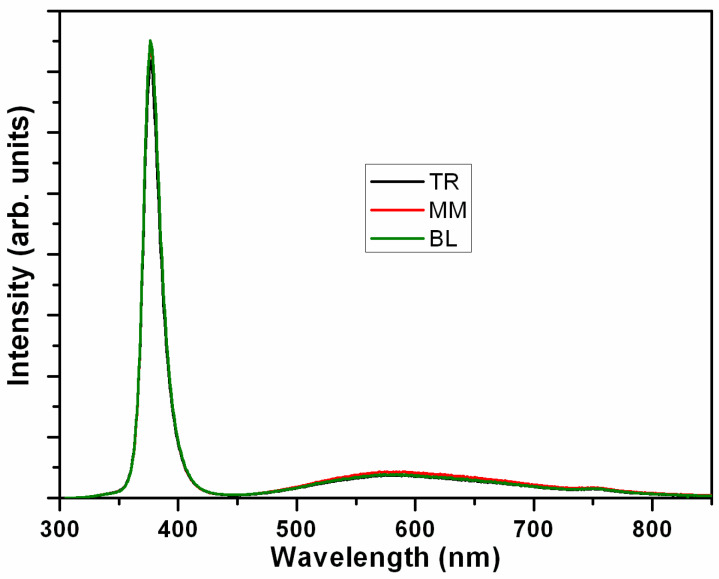
Room-temperature PL spectra of ZnO NRs in the TR, MM, and BL regions of sample 5.

By analyzing the XRF data, the average thickness of the ZnO, measured in atom units (a.u.) after the normalized XRF results, is determined to be 1.06, with a standard deviation (σ) of 0.07 a.u., as illustrated in Figure 3. The SEM results for the TR, MM, and BL regions are displayed in Figure 4. Top-view images indicate comparable homogeneity across different regions, and the enlarged insets reveal the hexagonal structure of the ZnO NRs. Cross-sectional micrographs are presented at the bottom of Figure 4, showing that the diameter of the NRs is 82 ± 5 nm. The density of these NRs is calculated to be (5.9 ± 0.2) × 10^9^ cm^−2^. The length of the NRs in the MM and BL regions is 480 ± 20 nm, while it is slightly longer in the TR region, measuring 510 ± 20 nm, aligning with the differences observed in the XRF results. Furthermore, the room-temperature photoluminescence (PL) spectra reveal a dominant and intense near-band-edge (NBE) emission at approximately 377 nm, consistent across different ZnO NR regions, as shown in Figure 5. Additionally, a weak broad defect emission in the visible spectrum and a near-infrared peak at approximately 750 nm are observed, as referenced in [47,48]. The optical quality of these large-scale as-grown ZnO NRs is noteworthy, and the PL results also indicate the homogeneity of the sample. These findings from sample 5 suggest that we can achieve relatively homogeneous NRs using the electrodeposition process.

**Figure 6:**
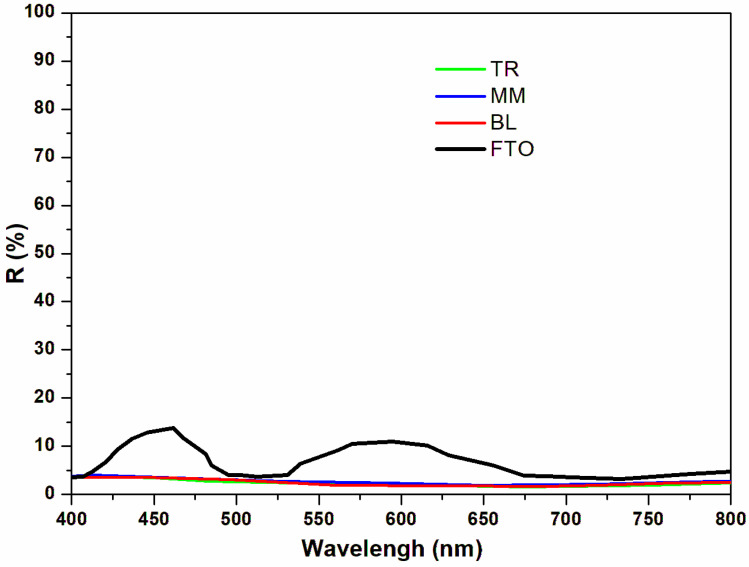
Reflectance spectra of FTO substrate without ZnO nanorods, and with ZnO nanorods in the TR, MM, and BL sections of sample 5.

To enhance our understanding of the impact of ZnO NRs as an antireflective coating (ARC) and to augment its light-trapping capabilities, we conducted an analysis of the wavelength-dependent reflectance of the sample (referred to as sample 5) both before and after the application of the ZnO NRs. This investigation spanned three distinct areas, as depicted in Figure 6. After the deposition of the ZnO NRs, a notable change was observed when compared with the FTO substrate. Specifically, the interference fringes, initially present, vanished post the growth of the ZnO NRs. Additionally, there was a significant reduction in the reflectance across a broad wavelength spectrum ranging from 400 to 800 nm. For comparison, we calculated the parameter of the averaged reflectance (Rave) without and with the ZnO NR ARCs, including the FTO sample and the TR, MM, and BL regions of sample 5, respectively, with the Air Mass 1.5 terrestrial global spectrum in the wavelength range of 400–800 nm, as given by [25]:

$$R_{ave}=\frac{\int F\left(\lambda \right)R\left(\lambda \right)d\lambda }{\int F\left(\lambda \right)d\lambda }$$

where *R*(*λ*) is the surface reflectance in the wavelength range of 400–800 nm, and *F*(*λ*) is the photon flux at Air Mass 1.5. The calculated solar average reflectance for the FTO and the three fabricated NR sections are 6.887%, 2.434%, 2.598%, and 2.267%, respectively. The average reflectance with the AM1.5 solar spectrum decreases significantly from 6.887% to 2.267–2.434%, so we can notice that the use of ZnO NRs as an antireflective layer on an FTO surface can significantly increase its light absorption.

This effect remained consistent across various sections of sample 5, namely the TR, MM, and BL sections, maintaining a similar spectral shape in these regions. Compared to FTO, the pronounced decrease in reflectance across this broad range can be attributed to the so-called ‘moth-eye effect’, which arises due to a continuously varying refractive index, leading to broadband suppression of reflection within the nanorod structure [49]. The theoretical analysis of this effect is elaborated upon and usually conducted using rigorous coupled-wave analysis (RCWA) [50]. The uniform antireflective property in different regions of the sample, demonstrated by the significant moth-eye effect in different regions of sample 5, also indicates the achieved homogeneity of the large-scale ZnO NR ARC.

Based on our experimental results and analysis, two primary factors influencing the homogeneity of large-scale zinc oxide nanorod (ZnO NR) electrodeposition are the solution flow and the electrical field. By appropriately adjusting the solution flow and the distribution of the electrical field, we can enhance the homogeneity of large-scale nanorods. This study further demonstrates that ZnO NRs are effective as an antireflective layer, offering potential for cost-effective, large-scale production and fabrication. Such advancements pave the way for increased industrial adoption and eventual commercialization, particularly in the realm of large-scale solar cells. The homogeneous, large-scale deposition of ZnO NRs as an antireflective coating (ARC) on solar cell surfaces significantly reduces sunlight reflection. This, in turn, enhances solar light absorption, boosting the photovoltaic conversion efficiency of these cells. Moreover, the ability of ZnO NRs to improve solar energy absorption makes them suitable for various heat collection applications, such as solar water heaters, greenhouses, etc. The outcomes of this research offer tangible benefits for advancing renewable clean energy resources, conserving conventional energy, and reducing carbon dioxide emissions. These findings underscore the potential of ZnO NRs in the field of green energy and highlight their role in promoting more sustainable energy practices.

## 4. Conclusions

We investigated the electrodeposition of ZnO NRs under varying solution flow states and using two sizes of Pt counter electrodes. Using experiments, we demonstrated that both the solution flow and the electric field significantly influence the homogeneity of large-scale ZnO NR electrodeposition. Utilizing a small counter electrode and stirring in the center, we successfully grew relatively homogeneous ZnO NRs on an 8 × 10 cm^2^ i-ZnO/FTO substrate. The photoluminescence (PL) results exhibited outstanding properties. The reflectance of the ZnO NRs, as examined in their reflectance spectra, was broadly suppressed across the wavelength region of 400–800 nm. We believe that by controlling the solution flow and adjusting the electric field distribution, larger and more homogeneous ZnO NRs with enhanced economic benefits can be achieved in future work. The optimized electrodeposited large-scale ZnO NRs show promise for applications in solar cells and heat collection systems, improving their light utilization efficiency. Additionally, this technology holds potential for LED applications, where ZnO NR ARCs fabricated on LED surfaces could improve the light output flux efficiency. Therefore, these prospective applications underscore the significance of this technology in energy conservation and emission reduction, making it worthy of further study and development.

## Figures and Tables

**Table 1:** Normalized XRF results of samples 1–3. Converting Zn content into ZnO thickness in testing NR region.

Sample	TL	TM	TR	ML	MM	MR	BL	BM	BR
1	1.46	1.1	1.54	1.21	1	1.30	1.47	1.06	1.43
2	1.41	1.15	1.42	1.30	1	1.36	1.30	1.07	1.34
3	1.29	1.11	1.30	1.18	1	1.13	1.16	0.95	1.16

**Table 2:** Normalized XRF results of samples 2 and 4. Converting Zn content into ZnO thickness in testing NR region.

Sample	TL	TM	TR	ML	MM	MR	BL	BM	BR
2	1.41	1.15	1.42	1.30	1	1.36	1.30	1.07	1.34
4	1.02	0.93	1.22	0.99	1	1.12	0.87	0.96	0.96

**Table 3:** Normalized XRF results of samples 5. Converting Zn content into ZnO thickness in testing NR region.

Sample	TL	TM	TR	ML	MM	MR	BL	BM	BR
5	1.16	1.11	1.12	1.06	1	1.11	0.99	0.95	1.02

## Data Availability

Data are contained within the article.
